# Association of microRNA-33a Molecular Signature with Non-Small Cell Lung Cancer Diagnosis and Prognosis after Chemotherapy

**DOI:** 10.1371/journal.pone.0170431

**Published:** 2017-01-31

**Authors:** Li-Kun Hou, Yu-Shui Ma, Yang Han, Gai-Xia Lu, Pei Luo, Zheng-Yan Chang, Ru-Ting Xie, Hui-Qiong Yang, Li Chai, Ming-Xiang Cai, Ting-Miao Wu, Fei Yu, Shan-Shan Qin, Zhong-Wei Lv, Chun-Yan Wu, Da Fu

**Affiliations:** 1 Department of Pathology, Shanghai Pulmonary Hospital, Tongji University School of Medicine, Shanghai, China; 2 Shanghai Engineering Research Center of Molecular Therapeutics and New Drug Development, College of Chemistry and Molecular Engineering, East China Normal University, Shanghai, China; 3 Department of Nuclear Medicine, Shanghai Tenth People’s Hospital, Tongji University School of Medicine, Shanghai, China; 4 Department of Radiology, Department of Translational Medicine Center & Medical Imaging Research Institute, Central Hospital of Baotou, Inner Mongolia, Inner Mongolia Medical University, China; 5 Veterinary Faculty, College of Veterinary Medicine, Hunan Agricultural University, Changsha, China; 6 Department of Pathology, Shanghai Tenth People’s Hospital, Tongji University School of Medicine, Shanghai, China; 7 Central Laboratory for Medical Research, Shanghai Tenth People's Hospital, Tongji University School of Medicine, Shanghai, China; University of North Carolina at Chapel Hill School of Medicine, UNITED STATES

## Abstract

**Objective:**

This study aims to explore the expression pattern and prognostic significance of *miR-33a* in non-small cell lung cancer (NSCLC) treated with adjuvant chemotherapy.

**Methods:**

*MiR-33a*expression in NSCLC was analyzed *in silico* using the GEO database and was subsequently confirmed by quantitative RT-PCR in 147 NSCLC biopsies. Among these, 32 of these biopsies were paired with adjacent non-neoplastic tissues. The survival analysis of NSCLC by Kaplan-Meier estimates was stratified based on *miR-33a* expression. In addition, multivariate survival analysis in corresponding groups of NSCLC patients was conducted by Cox proportional hazards regression model.

**Results:**

The *in silico* analysis of *miR-33a* expression in NSCLC resulted to its down-regulation in different tumor types. The expression level of *miR-33a* was lower in each grade of NSCLC tumor biopsies than in normal lung tissues. Univariate and multivariate survival analysis further established that low *miR-33a* expression was an important risk factor for overall survival and disease free survival in NSCLC patients.

**Conclusion:**

Our study implied that *miR-33a* expression levels may have an essential role in NSCLC progression, and could act as a specific and sensitive biomarker for NSCLC patients who have undergone adjuvant chemotherapy.

## Introduction

Lung cancer is the third most frequently diagnosed cancer and the leading cause of cancer-related mortality worldwide. There are approximately 1.8 million new lung cancer cases annually [1]. Patients with non-small cell lung cancer (NSCLC), which accounts for approximately 75–80% of the total lung cancer incidents, are mostly diagnosed at the advanced stages of the disease [2, 3]. For NSCLC patients who have undergone surgical resection, the American Society of Clinical Oncology (ASCO) guidelines recommend the use of post-operative therapeutic strategies including adjuvant external radiation therapy or cytotoxic chemotherapy combined with molecular targeted therapy [2, 4–7]. However, current staging approaches are inadequate in predicting and diagnosing the outcome of NSCLC treatments due to the unavailability of potential biomarkers for molecular targeted or personalized treatments. Therefore, improvement in molecular genetics diagnosis and the prediction of prognosis for targeted treatments and clinical decisions are urgently required.

MicroRNAs (miRNAs) are a large number of small noncoding RNA genes found to be aberrantly expressed in various types of malignancies and function either as oncogenes or tumor suppressors. This implies that miRNAs play a vital role in tumorigenesis and cancer progression [8–11]. Furthermore, these have also been shown to be involved in oncogenesis mechanisms, which can serve as potential cancer biomarkers [12]. Therefore, the understanding of miRNA expression patterns as potential biomarkers for the diagnosis and prognosis of personalized targeted therapies and clinical decision and management has just started to unfold [12–15]. The identification of a miRNA signature that can predict the benefit from adjuvant chemotherapy would be definitely helpful for the clinical decision and management of NSCLC in patients. However, it remains ambiguous whether the miRNA signature can predict the clinical decisions of NSCLC, including major adjuvant chemotherapy or TNM stage.

Although a majority of patients are diagnosed initially by imaging techniques, the prognosis of patients with this gene mutation remains poor in traditional treatment. Fluorodeoxyglucose (FDG)-PET/CT scans play an important role not only in the staging of lung cancer, but also in predicting and assessing treatment responses at the present time. However, these could not provide genetic information useful for predicting adjuvant chemotherapeutic or gene targeting therapeutic options. The use of genomics-based diagnosis for patients with resectable tumors and the combination of chemotherapy, radiation therapy in conjunction with molecular targeted therapy, would improve overall survival (OS) and disease-free survival (DFS) in patients with locally advanced lung cancer. These miRNAs have the potential to regulate the expression of thousands of corresponding target genes, and are able to govern a comprehensive range of biological functions such as cellular proliferation, differentiation, apoptosis, immune response, and the maintenance of cell and tissue identity [16, 17]. The results of molecular exploration may improve clinical decisions and management for NSCLC patients [18]. Advances in genomics, transcriptomics and proteomics have resulted in the generation of many candidate biomarkers with potential clinical significance.

There has previously reported that low levels of miR-33a expression were found in NSCLC patients in clinical and suggested that the *miR-33* family might play a significant role in NSCLC prognosis and patient survival [19]. However, few are known on the associations between *miR-33* levels and NSCLC patients treated with adjuvant chemotherapy. Therefore, in order to investigate whether specific and sensitive biomarkers can predict the clinical outcome of NSCLC at the molecular level, including the prognosis and response to adjuvant chemotherapy, we focused on the role of miRNAs, and attempted to determine a link between its expression and NSCLC survival.

## Material and Methods

### Ethics statement

The study was approved by the Ethics Committee of Shanghai Tenth People’s Hospital, Tongji University School of Medicine (SHSY-IEC-pap-15-18). Each participant provided a written informed consent before participating in this study. All specimens were handled and made anonymous according to ethical and legal standards.

### GEO data acquisition and processing

*MiR-33* expression in NSCLC biopsies was analyzed *in silico* using the GEO database. The GEO database provides a multimodal data repository and retrieval system for high-throughput functional genomic data generated by microarray and next-generation sequencing technologies and can be acquired from the GEO website (The Gene Expression Omnibus, http://www.ncbi.nlm.nih.gov/geo/) [20, 21].

### Acquisition of clinical specimens

Fresh frozen tissue samples from NSCLC patients, who underwent surgical resection between 2008 and 2012, were obtained from the tissue bank of Shanghai Tenth People’s Hospital. These samples included paired tumor and adjacent non-cancerous tissues (n = 32), as well as a large cohort of individual NSCLC biopsies (n = 115). The histological typing of these tumors was performed according to the World Health Organization criteria. Staging was performed according to the Seventh Edition of the American Joint Commission on Cancer (AJCC) tumor-node-metastasis (TNM) staging system for NSCLC [22], and patient data was collected up to May 30, 2015. The clinical information recorded included the patient’s characteristics, tumor characteristics, OS, DFS and chemotherapy status.

### RNA isolation and the detection of miR-33a expression by qRT-PCR

Total RNA was extracted from NSCLC and normal tissues using TRIZOL reagent, according to manufacturer’s instructions. RNA concentration was measured by a spectrophotometer, and the quality of all RNA samples was assessed by electrophoresis on 1.5% denaturing agarose gels. qRT-PCR was carried out using a Taqman miRNA PCR kit (Applied Biosystems, Foster City, CA) according to manufacturer’s instructions. Briefly, total RNA was reverse-transcribed to cDNA using AMV reverse transcriptase (TaKaRa, Dalian, China) and the stem- loop RT primers (Applied Biosystems). Real-time PCR was performed using TaqMan miRNA probes on the Applied Biosystems 7300 Sequence Detection System (Applied Biosystems). U6 was used as the internal control. The 2^-δδ^CT method was used to quantify the expression levels of *miR-33a*, and the expression status (e.g., high levels or low expression levels) was recorded.

### Statistical analysis

All statistical analyses were accomplished with IBM SPSS statistics software Version 20.0 for Windows. The expression of *miR-33a* was represented as the mean ± standard deviation. Independent *t*-test was used to examine the differences between two groups and Chi-square test was used to evaluate the differences in rates between groups. Kaplan-Meier curves were used to determine the OS of various groups, and results were compared using the log-rank test. Univariate and multivariate survival analyses were based on the Cox regression model, and this model was used to identify the independent factors that had significant effects on survival. A *P-*value *<* 0.05 was considered statistically significant.

## Results

### Analysis of *miR-33a* expression in cancer using the *in silico* data platform

**Fig 1:**
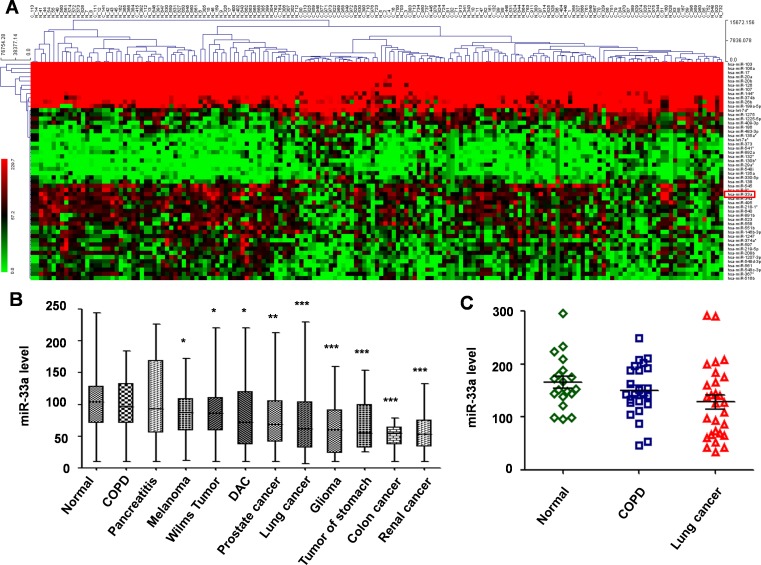
Analysis of *miR-33a* expression from GEO datasets. A, Data from the GEO dataset (GSE59153) was clustered using MEV 4.7.1 software. B, Relative expression levels of *miR-33a* in different cancers *vs*. normal controls from the GEO dataset (GSE59153). C, Relative expression levels of *miR-33a* in NSCLC and adjacent normal tissues from patients with cancerous and non-cancerous lung diseases from the GEO dataset (GSE24709).

Microarray description and raw data have been made available in the GEO database with reference number GSE59153. This project analyzed the peripheral blood profiles of patients from various cancers (diseases), and controls. These included normal controls (n = 94), lung cancer individuals (n = 71) and patients (n = 940) have been screened for complete miRNA (1,049 miRNAs) repertoire, according to miRNAse V12-14. Each miRNA was measured in seven replicates at least, and the median of the replica was computed. The *in silico* data sample hierarchal clustering of the gene expression microarrays data was performed using the MEV 4.7.1 clustering software. The 52 miRNAs with significant changes (*miR-33a* included, *P* < 0.001, Fold change (FC) ≥ 2 or ≤ 0.5) were filtered out, and following the expression of differentially expressed miRNAs was assessed with reference to the prognosis of the NSCLC patient (Fig 1A). Among the 52 miRNAs from the GEO database, 19 miRNAs were up-regulated and 33 miRNAs were down-regulated. This included previously published up-regulated miRNAs in lung cancer, such as miR-130b*, miR-135a and miR-138, and down-regulated miRNAs, such as miR-126, miR-144, miR-20a/b and miR-218-1 [19].

After ruling out these known differentially expressed miRNAs in NSCLC, the rest of the differentially expressed miRNAs in normal, lung cancer, chronic obstructive pulmonary disease (COPD), and pancreatitis with different types of tumors (disease) were further analyzed; and *miR-33a* was the only one found to be significantly down-regulated in all the different tumor types (Fig 1B).

In addition, we also downloaded peripheral profiles from patients with cancerous and non-cancerous lung diseases (including 19 normal controls, 28 lung cancer patients and 24 COPD samples) (GSE24709), and found that also *miR-33a* was significantly reduced in lung cancer (*P =* 0.035; Fig 1C).

However, we noticed that *miR-33a* was reported to highly express in tumor samples from glioma, gastric, renal and colon cancer [23–25]. Thus, in our current study, we were particularly interested in *miR-33a* expression and clinical significance to NSCLC prognosis.

### Analysis of *miR-33a* expression in NSCLCs and normal lung tissues by qRT-PCR

**Fig 2:**
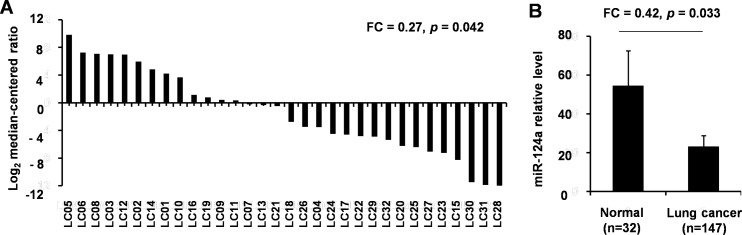
Expression of *miR-33a* in normal lung and NSCLC tissues. A, The expression levels (log_2_ median-centered ratio) of *miR-33a* in 32 paired cancers *vs*. adjacent non-tumor tissues were analyzed. B, The *miR-33a* expression in NSCLC samples (*n* = 147) and paired adjacent non-tumor tissues (*n* = 32) was analyzed.

In order to further confirm the results of the *in silico* gene microarray study, we analyzed the *miR-33a* expression in large samples from clinic. A total of 147 (94 grade I-II, 52 grade III-IV) tumor specimens and 32 normal lung specimens were included for this analysis. The 32 samples had paired tumor and adjacent non-cancerous tissues (n = 32). The *miR-33a* analysis in these paired samples by qRT-PCR demonstrated that its expression levels were significantly lower in NSCLC tumor biopsies relative to adjacent non-neoplastic tissues. This difference was statistically significant (*P =* 0.042, FC = 0.27; Fig 2A). Moreover, the analysis of other tumor specimens also revealed that the level of *miR-33a* expression was significantly lower in each grade of NSCLC tumor biopsy than in normal lung tissues (*P =* 0.033, FC = 0.42; Fig 2B).

### Correlation of *miR-33* expression with demographic factors and clinical characteristics

In order to validate whether *miR-33a* expression levels were affected by clinical characteristics, we explored the correlation of *miR-33a* expression with demographic and clinical factors. All 147 patients of NSCLC included in this study demonstrated that *miR-33a* expression levels were negatively correlated with lymph-node metastasis (*P =* 0.028), TNM stage (*P =* 0.038), invasion of the lung membrane (*P =* 0.039) and the diameter of the tumor (*P =* 0.027) as shown in Table 1. However, we did not observe any association between *miR-33a* expression and patient gender, age, smoking history, tumor differentiation, histology, vascular invasion and adjuvant chemotherapy (*P >* 0.05, Table 1).

**Table 1:** Univariate analysis of overall survival in NSCLC patients stratified based on clinical characteristics.

Factor	Variable	N	*miR-33a* expression (Median)	P value	Overall survival	
					Months (Median)	95% CI (Mean)
Age	≥ 60	92	29.24	0.121	32.26	29.32–35.21
	< 60	55	18.75		32.02	29.12–34.93
Gender	Male	89	23.1	0.928	32.11	29.27–34.94
	Female	58	22.09		35.87	33.01–38.74
Smoking history	Never	35	24.01	0.118	32.05	28.74–29.16
	Ever	41	19.18		30.25	27.43–35.06
	Unknown	71	26.54		30.88	25.39–33.48
Lymph-node metastasis	Negative	85	27.06	**0.028**	31.28	24.72–33.87
	Positive	50	14.14		29.34	26.43–32.58
	Unknown	12	18.73		30.26	28.34–33.16
Tumor differentiation	Poorly	51	18.96	0.051	28.93	26.54–30.65
	Moderately	88	25.43		29.33	27.06–31.54
	Well	8	26.08		31.69	29.64–33.71
Histology	Adenocarcinoma	94	24.63	0.654	28.43	26.55–31.29
	Squamous cell carcinoma	52	26.07		32.38	28.43–35.66
TNM stage	I-II	94	28.09	**0.038**	33.58	30.19–35.48
	III-IV	52	22.14		27.68	24.36–29.13
Invasion of lung membrane	Negative	30	23.35	**0.039**	32.67	29.89–34.26
	Positive	105	16.75		28.03	26.74–30.52
	Unknown	12	18.94		29.31	27.65–35.43
Vascular invasion	Negative	132	23.16	0.652	30.39	28.45–32.33
	Positive	9	19.03		29.04	26.71–31.31
	Unknown	6	20.36		28.33	26.58–31.46
Chemotherapy	Negative	74	22.35	0.557	26.94	23.06–28.94
	Positive	62	21.03		32.47	29.64–34.53
	Unknown	11	23.07		28.03	26.54–32.19
Diameter	≥ 5 cm	37	17.43	**0.027**	24.86	25.39–30.01
	< 5 cm	110	26.03		32.43	31.21–34.68

### Univariate analysis of OS in NSCLC patients stratified based on clinical characteristics

A univariate survival analysis was performed through Kaplan-Meier estimates by stratifying NSCLC patients based on clinical factors (including gender, age, smoking history, lymph-node metastasis, tumor differentiation, histology, TNM stage, invasion of the lung membrane, vascular invasion, and tumor size). Median follow-up was 39.6 months (range: 14.6 to 89.6 months). Results of the univariate analyses are shown in Table 1.

As expected, there was a significant association between shorter OS and classical prognostic factors such as lymph-node metastasis (*P =* 0.045), TNM stage (*P =* 0.012), invasion of the lung membrane (*P =* 0.018), and tumor size (≥ 5 cm, *P*< 0.001). In addition, the univariate analysis using the Cox proportional hazards regression model revealed that lymph node metastasis (*P =* 0.04, HR = 1.54 [1.16, 1.86]), TNM stage (*P =* 0.01, HR = 2.29 [2.72, 2.38]), invasion of the lung membrane (*P =* 0.02, HR = 1.76 [1.45, 1.99]) and diameter (*P =* 0.001, HR = 2.56 [2.03, 3.45]) were positively correlated with poorer prognosis (Table 2).

**Table 2:** Cox regression model analysis for prognosis based on various clinical characteristics of NSCLC patients.

Factor	HR	95% CI (univariate)	*P* value	*miR-33a* multivariate analysis		
				HR	95% CI (multivariate)	*P* value
Age	0.95	0.71–1.15	0.78			
Gender	0.88	0.54–1.26	0.36			
Smoking history	1.22	0.73–1.38	0.12			
Lymph-node metastasis	1.54	1.16–1.86	**0.04**	1.78	1.20–2.66	**0.02**
Tumor differentiation	0.78	0.54–1.11	0.16			
Histology	1.19	0.88–1.24	0.11			
TNM stage	2.29	2.72–3.38	**0.01**	2.43	1.86–2.98	**0.008**
Invasion of lung membrane	1.76	1.45–1.99	**0.02**			
Vascular invasion	0.91	0.68–1.24	0.75			
Diameter (≥ 5 cm)	2.56	2.03–3.45	**0.001**	3.17	2.26–5.03	**<0.001**
Low *miR-33a* expression	1.66	1.21–2.24	**0.028**			

**Fig 3:**
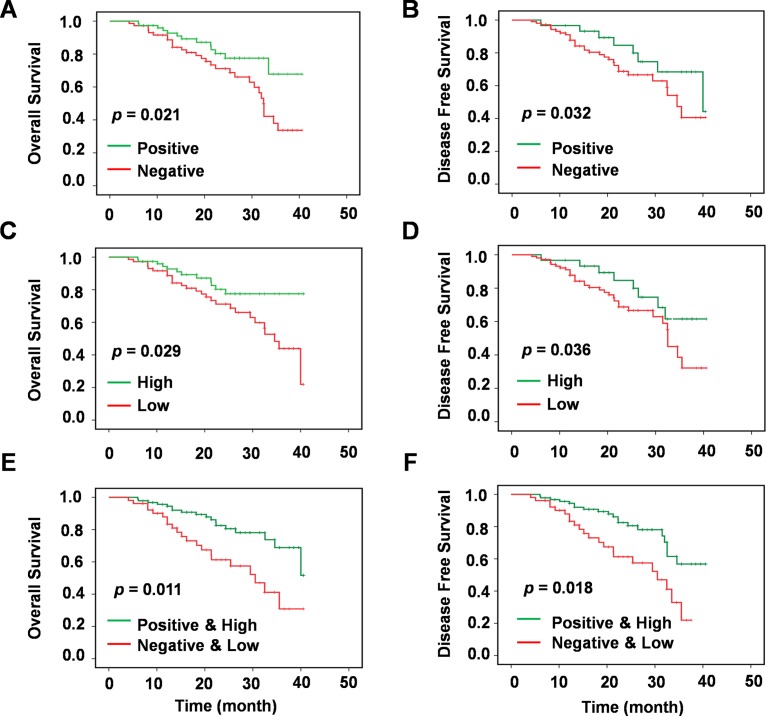
Survival analysis of *miR-33a* expression and chemotherapy in NSCLCs. Univariate survival analysis of overall survival and disease-free survival in lung carcinoma as determined by Kaplan-Meier plots estimates based on chemotherapy (Negative *vs*. Positive) in (A) and (B), *miR-33a* expression (Low *vs*. High) in (C) and (D), and *miR-33a* expression and chemotherapy (non-chemotherapy and low miR-33a expression *vs*. chemotherapy and high miR-33a expression) in (E) and (F), respectively.

In order to assess the influence of chemotherapy on the prognosis of NSCLC patients, Kaplan-Meier survival curves were plotted and log rank analysis was performed. The results revealed that adjuvant chemotherapy was significantly associated with increase OS (*P =* 0.021, Fig 3A) and DFS (*P =* 0.032, Fig 3B) in NSCLC patients.

Thus, significantly reduced OS was associated with patients who had not undergone adjuvant chemotherapy or had lymph node metastasis, invasion of the lung membrane, or increased tumor size (≥ 5 cm).

### Assessment of *miR-33a* as a prognostic tumor-marker for NSCLC patient survival

Results of the survival analysis demonstrated that patients with low *miR-33a* expression levels had poor OS rates compared to patients with high *miR-33a* expression. The low expression of *miR-33a* was significantly associated with decreased OS (*P =* 0.029, Fig 3C) and DFS (*P =* 0.036, Fig 3D) in NSCLC patients. Similarly, the low expression of *miR-33a* was also positively correlated with poor prognosis (*P =* 0.028, HR = 1.66 [1.21, 2.24]) (Table 2).

In order to further assess the contribution of other variables with *miR-33a* as a prognostic marker in NSCLC patients, we also performed a multivariate survival analysis, using the Cox proportional hazards regression model. This analysis initially included all parameters that were predictive of OS in the univariate analysis of the entire study group, as presented in Table 2 (age, gender, smoking history, lymph-node metastasis, tumor differentiation, histology, vascular invasion and diameter, and invasion of the lung membrane). A forward stepwise procedure was adopted to obtain the final model of significant predictors for OS, which consist of factors including lymph-node metastasis, diameter, TNM stage, and expression of *miR-33a*. According to this analysis, low *miR-33a* expression was identified as a predictor of shorter OS in NSCLC patients (Table 2).

### Analysis of the predictive value of *miR-33a* expression for adjuvant chemotherapy

As adjuvant chemotherapy provides primary treatment after surgical operations in majority of NSCLC cases, the OS and DFS of patients were consequently explored based on the treatment signature. Adjuvant chemotherapy was identified to be significantly associated with increased OS (*P =* 0.021, Fig 3A) and DFS (*P =* 0.032, Fig 3B) in patients in this cohort. The Kaplan-Meier univariate and multivariate Cox proportional hazards regression survival analysis was further conducted to determine whether adjuvant chemotherapy and/or *miR-33a* expression were associated with OS and DFS. When this adjuvant chemotherapy data was analyzed based on the expression *of miR-33a*, OS and DFS were both observed to be significantly longer in NSCLC patients with high *miR-33a* expression as opposed to patients who were untreated or with a low expression (*P =* 0.011 and 0.018, respectively) (Fig 3E and 3F), which suggests that NSCLC patients with lower *miR-33a* expression has poor prognosis even when treated with adjuvant chemotherapy.

## Discussion

The identification of specific and sensitive tumor-markers for revealing human malignancies is urgently required to decrease the global morbidity and mortality rate caused by cancer [26, 27]. With the effort of identifying ideal cancer markers, qRT-PCR methodologies have been extensively explored [15, 28]. In this study, we have established an effective strategy that allowed us to identify miRNA-based biomarkers for NSCLC. NSCLC biopsies and adjacent non- cancerous tissues were assessed for *miR-33a* expression. The expression of *miR-33a* was observed to be down-regulated in NSCLC patients. In this large sample population, we observed that the elevated expression of *miR-33a* was associated with the better prognosis of NSCLC patients who have undergone cytotoxic chemotherapy; which was consistent with a previously published study. Gong *et al*.[29] stated that *miR-33a* was expressed at lower levels in metastatic NSCLC cells. In this study, qRT-PCR was conducted to measure *miR-33a* expression levels in 53 pairs of NSCLC tumor and non-tumor tissue samples, and results demonstrated that the low-expression of *miR-33a* was predictive of poor prognosis in NSCLC patients [29]. In another study, the use of *miR-33a* was validated as a novel therapeutic target for colon carcinoma in a mouse model of preclinical study [24]. Therefore, based on these results it is reasonable to indicate that *miR-33a* expression levels may play a critical role in NSCLC progression; which could develop as a promising, specific and sensitive diagnostic biomarker for NSCLC patients at advanced stages. This also implies that *miR-33a* may be a novel and valuable tumor-marker, and increasing the cellular levels of *miR-33a* may be a novel therapeutic strategy for the treatment of patients with advanced NSCLC.

In general, human cancers that are comparable other diseases, are easier to treat and control when revealed at the early stage of disease progression [30, 31]. Distant metastasis usually triggers more than 80% of cancer deaths and involves a complicated sequence of steps where cancer cells leave the original position and migrate to other segments of the body through the circulatory and lymphatic system [32, 33]. Qi *et al*. [34] revealed that *miR-33a* expression is very poor in extreme metastatic breast cancer cell lines than noncancerous breast epithelial cells and non-metastatic breast cancer cells. At the same time, *miR-33a* has also been shown to inhibit breast cancer cell growth, migration and invasion together with the suppression of *in vivo* tumor growth and the lung metastasis of breast cancer cells [34]. Ke*et al*. [35] also described that human metastatic melanoma cells have low *miR-33a/b* expression and is involved in the regulation of *in vivo* functions by acting as a tumor suppressor through targeting HIF-1α. The identification of this miR-33a/HIF-1α axis has been proposed to be a novel approach for the management of melanoma. [35]. Tumor recurrence and metastasis is crucial in patients not only under treatment with traditional chemotherapies, but also with more current molecular targeted therapies [18, 36]. The identification of miRNA-based markers for various types of cancers could help molecular based cancer classification [28, 37]. The important role of these miRNAs have been shown to play a role in cancer by targeting various signaling pathways and therapeutic responses to the target gene has delivered a new opening for developing novel agents and methodologies [38].These above results revealed that *miR-33a* could be used as a prospective therapeutic RNA mimic for the treatment of patients with metastasis/advanced carcinoma. Thus, it is necessary to screen miRNAs in a genome-wide manner, and determine all differentially expressed miRNAs in NSCLC patients for the guiding clinical management and use of adjuvant chemotherapy. The emergence of the association between *miR-33a* expression and chemotherapy from our study suggests a promising role for this biomarker in response to chemotherapy.

However, not all published studies suggest a positive association between *miR-33a* levels and cancer-relative diagnosis and therapy. *MiR-33a* level has been shown to be negatively correlated with the target gene TWIST and was over-expressed in chemo-resistant osteosarcoma; which resulted in the down-regulation of TWIST, and increased osteosarcoma cell resistance to cisplatin [39]. It was suggested that inhibition of miR-33a/TWIST signaling could be a latent novel approach to develop neoadjuvant chemotherapy for osteosarcoma. Moreover, understanding the molecular features of lung malignancies would help in targeted therapy development.

Although contemporary findings on the link between NSCLCs and miRNAs have drastically stretched our realization on the signaling pathway and its association in the pathogenic developments of NSCLC, our understanding of underlying mechanisms that integrate the activity of the molecular pathway remains incomplete. An extensive search on the regulatory roles of miRNAs in the signaling pathway may contribute to the possible option of using miRNAs as predictive, specific and sensitive tumor-markers.

### Conclusion

In conclusion, our data confirmed that with adjuvant chemotherapeutic treatment, the median OS and DFS of patients improved. Furthermore, the analyses firstly demonstrated that the high expression of *miR-33a*, together with adjuvant chemotherapy, greatly improved OS and DFS in NSCLC patients. Particularly, the application of *miR-33a* as specific and sensitive biomarkers may also be useful for predicting therapeutic responses in advanced NSCLC patients, which could lead to a superior level of personalized therapy. Thus, *MiR-33a* can be regarded as a potential tumor biomarker for chemosensitivity in NSCLC patients.
